# Supplementary material for: Surpassing spectator qubits with photonic modes and continuous measurement for Heisenberg-limited noise mitigation
Source: npj Quantum Inf. 2023 Aug 11;9(1):81. doi: 10.1038/s41534-023-00748-y (PMC11080661; doi:10.1038/s41534-023-00748-y)
Supplement: Supplementary file 1 — Supplementary Information [file 41534_2023_748_MOESM1_ESM.pdf]

Supplementary Information: Surpassing spectator qubits with photonic modes and continuous measurement for Heisenberg-limited noise mitigation

Andrew Lingenfelter[1,2*] and Aashish A. Clerk[1]

[1] *Pritzker School of Molecular Engineering, University of Chicago, Chicago, IL 60637, USA*

[2] *Department of Physics, University of Chicago, Chicago, IL 60637, USA*

[*] lingenfelter@uchicago.edu

(Dated: July 12, 2023)

I. SUPPLEMENTARY METHODS

Feedforward delay. Delay in the measurement and feedforward will degrade the noise mitigation performance of the spectator. To include delay in the measurement and feedforward, we use the fact that the measurement is classical and nonreciprocal. The classicality implies that the qubit and spectator never become entangled; thus from the perspective of the qubit, the spectator mode is another classical noise source that happens to be correlated with $\xi_q(t)$. The nonreciprocity implies that there is no feedback from the qubit to the spectator; thus there is no way for the qubit to learn about the feedforward delay. Together these imply that from the perspective of the qubit, a delay in the measurement and feedforward, is equivalent to instantaneous measurement and feedforward of a delayed noise signal. We therefore model the feedforward delay as a detection delay: at time t, the spectator detects delayed noise $\xi_s(t-\tau_d)$ instead of the instantaneous noise $\xi_s(t)$. The delayed signal is then instantaneously fed forward to the qubit.

Due to the delay, the spectator transduction factor α_s picks up a phase factor

$$\alpha_s \mapsto e^{-i\omega\tau_d}\alpha_s \tag{1}$$

which reduces the spectator mode's ability to mitigate noise within its detection bandwidth. In the long-time limit, however, the qubit remains sensitive only to the zero-frequency noise, thus the long-time dephasing rate vanishes for $\alpha_s = 1$.

The effects of delay are best illustrated with an example. We consider a white noise spectral density $S[\omega] = S_0$ and a delay τ_d. The decoherence function is

$$\chi(t) = \Lambda_{imp}(t) + \begin{cases} S_0 t + \frac{S_0}{\kappa_\phi}\left(e^{-\kappa_\phi t/2} - 1\right) & t < \tau_d \\ S_0\tau_d + \frac{S_0}{\kappa_\phi}\left(1 - 2e^{-\kappa_\phi (t-\tau_d)/2} + e^{-\kappa_\phi t/2}\right) & t > \tau_d \end{cases} \tag{2}$$

where $\Lambda_{imp}(t)$ is still given by Eq. (26) of the main text. For pre-delay times $t < \tau_d$ the spectator feedforward noise is uncorrelated with the direct noise on the qubit, leading to the initial dephasing at twice the bare rate, $S_0 t$. There is also an exponential decay to a constant due to the Lorentzian spectral density of the noise fed forward from the spectator (bandwidth κ_ϕ). In the long-time limit the dephasing due to $\xi(t)$ is constant in time:

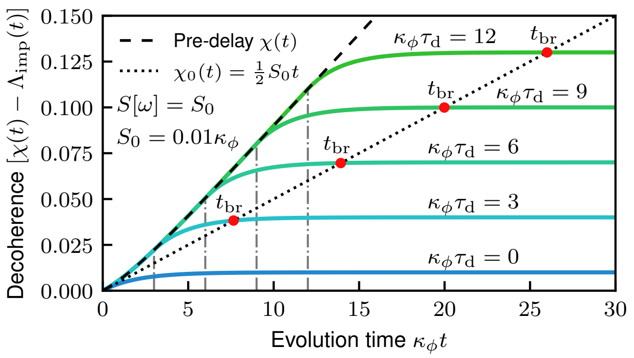

Supplementary Figure 1. **Effects of feedforward delay on qubit decoherence.** The qubit decoherence function (less $\Lambda_{imp}(t)$) vs. time for various indicated delays τ_d and for white noise $S[\omega] = S_0$ with $S_0 = 0.01\kappa_\phi$. The dotted line is the bare qubit decoherence function and its intersection with the delay curves denotes the minimum break-even time t_{br} for that delay τ_d. The dashed "pre-delay $\chi(t)$" curve is given by Eq. (2) evaluated as though $t < \tau_d$ for all times, and the vertical dot-dashed lines indicate the delay time $\kappa_\phi\tau_d$ for each delay curve – this is the time when the spectator begins mitigating noise.

$$\chi(t \to \infty) = \Lambda_{imp}(t) + S_0\tau_d + \frac{S_0}{\kappa_\phi} \tag{3}$$

where the constants are the delay-dependent dephasing ($S_0\tau_d$) and the zero-delay initial dephasing ($\chi_{init}(\infty) = S_0/\kappa_\phi$, cf. Eq. (37) of the main text).

The qubit decoherence function with feedforward delay is shown in Supplementary Fig. 1 for white noise. The bare dephasing of the qubit $\chi(t) = S_0 t/2$ intersects each delay dephasing function at the minimum possible the break-even time t_{br}: the time at which the spectator system improves over the bare decoherence assuming negligible $\Lambda_{imp}(t_{br}) \ll S_0 t_{br}$. For $\tau_d \gtrsim 1/\kappa_\phi$, the minimum break-even time is $t_{br} \approx 2(\tau_d + 1/\kappa_\phi)$, and for $\tau_d \ll 1/\kappa_\phi$ the minimum break-even time vanishes as $t_{br} = (2+\sqrt{2})\tau_d$.

Internal loss and optimal squeezing. Throughout the main text, we assume that the coupling rate of the spectator to the output waveguide is the domi-

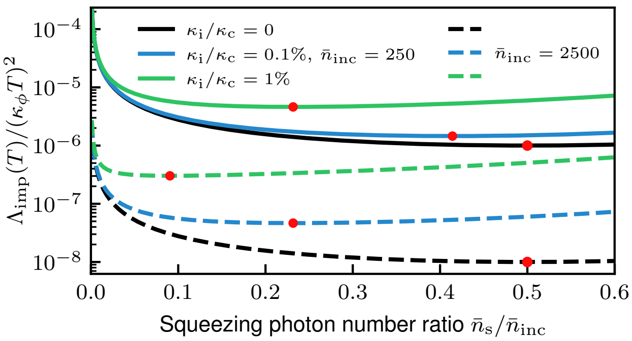

Supplementary Figure 2. **Effects of spectator mode internal loss on the optimal ratio of temporal mode squeezing photons.** $\Lambda_{imp}(T)/(\kappa_\phi T)^2$ (cf. Eq. (7)) vs. squeezing photon number ratio $\bar{n}_s/\bar{n}_{inc}$ in the presence of indicated degrees of internal loss κ_i/κ_c, for a fixed long evolution time $T \gg 1/\kappa_\phi$ and $g = 1$. We show $\bar{n}_{inc} = 250$ in solid lines and $\bar{n}_{inc} = 2500$ in dashed lines; the colors denote various κ_i/κ_c. The minimum of each curve is marked by a red dot and denotes the optimal fraction of the total photon number which should be used in the squeezing to minimize the long-time dephasing rate. Note that for a given κ_i/κ_c, the optimal squeezing photon number ratio decreases for increasing $\bar{n}_{inc}$; this is the breakdown of Heisenberg-limited scaling with internal loss.

nant source of damping and any internal loss is extremely weak: $\kappa_i \ll \kappa_c$. Internal loss is experimentally unavoidable, however, and it effectively acts as a second pathway for the feedforward signal to leak out of the spectator. This additional signal sink is most clearly an issue when the spectator is strongly squeezed as it limits how much the vacuum noise in the output field can be reduced.

We let the mode couple to a zero temperature internal loss bath (although this can be generalized to finite temperature in a straightforward manner) with rate κ_i. The total loss rate is

$$\kappa_{tot} = \kappa_c + \kappa_i \tag{4}$$

The drive strengths must be increased in proportion to the damping rate; the overall rate scale κ_c in the drive Hamiltonian (cf. Eq. (5) of the main text) is replaced by $\kappa_c \mapsto \kappa_{tot}$. The ideal spectator transduction factor is now

$$\alpha_s^{ideal} = \frac{\kappa_{tot}}{\kappa_c} \geq 1, \tag{5}$$

and all of the properties of the spectator at ideal transduction strength otherwise hold.

The internal loss effects a qualitative change to the measurement imprecision noise dephasing Λ_{imp}:

$$\Lambda_{imp}(t) = \frac{\alpha_s^2}{32\beta_s^2 \bar{n}_1} \frac{\kappa_c}{\kappa_{tot}} \left[\frac{(1-\lambda_2)^2}{1+\lambda_2} + \frac{\kappa_i}{\kappa_c}(1+\lambda_2) \right] \kappa_\phi t. \tag{6}$$

Here we neglect the exponential decay to a constant. These terms each receive a $(\kappa_c/\kappa_{tot})^2$ prefactor, and the exponential gets the replacement $\kappa_\phi \mapsto \kappa_{tot,\phi}$. The linear-in-$t$ term is no longer simply proportional to $(1-\lambda_2)^2$. The term $\propto \kappa_i$ is caused by the splitting of the mode's output field between the internal loss and the waveguide, which limits the degree of squeezing in the waveguide.

In terms of the number of photons in the temporal mode $\hat{A}$ (cf. Eq. (43) of the main text), and for $\bar{n}_{inc}, \bar{n}_d, \bar{n}_s \gg 1$, the long-time measurement imprecision noise dephasing is now given by

$$\Lambda_{imp}(T) = \frac{(\kappa_\phi T)^2}{64\beta_s^2} \frac{\kappa_{tot}}{\kappa_c} \left[\frac{1}{\bar{n}_d \bar{n}_s} + \frac{2\kappa_i/\kappa_c}{\bar{n}_d} \right] \tag{7}$$

where we have let $\alpha_s = \kappa_{tot}/\kappa_c$, its ideal value. For a fixed $\bar{n}_{inc}$ in the temporal mode, the optimal choice of $\bar{n}_d$ and $\bar{n}_s$ is no longer $\bar{n}_d = \bar{n}_s = \bar{n}_{inc}/2$, but is now dependent on the internal loss ratio κ_i/κ_c, as we show in Supplementary Fig. 2. The optimal choice rapidly approaches $\bar{n}_d = \bar{n}_{inc}$ when the product $\bar{n}_{inc}(\kappa_i/\kappa_c) \gg 1$. Furthermore the Heisenberg-limited scaling $\Lambda_{imp}(T) \propto 1/\bar{n}_{inc}^2$ rapidly breaks down as the internal loss term in Eq. (7) becomes dominant with increasing $\bar{n}_{inc}$ because it is $\propto \bar{n}_d \sim 1/\bar{n}_{inc}$ instead of $\sim 1/\bar{n}_{inc}^2$.
